# New Insights into the Phenology and Overwintering Biology of *Glyptapanteles porthetriae*, a Parasitoid of *Lymantria dispar*

**DOI:** 10.3390/insects16121270

**Published:** 2025-12-14

**Authors:** Thomas Zankl, Christa Schafellner

**Affiliations:** Institute of Forest Entomology, Forest Pathology and Forest Protection, Department of Ecosystem Management, Climate and Biodiversity, BOKU University, AT-1190 Vienna, Austria

**Keywords:** winter dormancy, endoparasitoid, Braconidae, Erebidae, spongy moth, hyperparasitism

## Abstract

*Glyptapanteles porthetriae* (Hymenoptera: Braconidae) is a solitary larval endoparasitoid of the spongy moth, *Lymantria dispar* (Lepidoptera: Erebidae). Previous studies suggested that only the spring generation of the wasp parasitizes early instars of *L. dispar*, while subsequent generations develop in larvae of unidentified alternative hosts and overwinter exclusively as endoparasitic larvae within these hosts. In a declining outbreak population of *L. dispar* in Austria, we observed two successive generations of *G. porthetriae* developing in spongy moth larvae during spring and early summer. The high abundance of parasitoids in the third outbreak year prompted us to test alternative hypotheses proposing a univoltine, monophagous life cycle with strong specialization on *L. dispar*. We exposed various developmental stages of *L. dispar* embryonic larvae to wasp females and evaluated potential dormancy induction in *G. porthetriae* larvae or pupae by assessing developmental parameters and respiratory activity under different environmental conditions. *G. porthetriae* did not develop from *L. dispar* individuals exposed in the egg stage, and no dormancy was observed in *G. porthetriae* developing in *L. dispar* larvae. These findings disprove the univoltine, monophagous development hypotheses and highlight the reliance of *G. porthetriae* on suitable alternative hosts to complete its life cycle.

## Simple Summary

The braconid wasp *Glyptapanteles porthetriae* is a key natural enemy of the spongy moth, *Lymantria dispar*, a major oak forest defoliator. The wasp larvae develop inside caterpillars, ultimately causing the host’s death. Literature suggests that *G. porthetriae* primarily parasitizes young spongy moth larvae in spring. Its offspring emerge in early summer and parasitize unidentified alternative host species, where the wasp larvae remain through autumn and winter. Alternative hypotheses propose a stronger specialization on *L. dispar*, with the wasps overwintering in spongy moth egg larvae or inside their own cocoons. These hypotheses were supported by unexpectedly high parasitization rates observed in an Austrian spongy moth field population during the final year of an outbreak, when moth abundance far exceeded the availability of alternative hosts. This study highlights unique aspects of *G. porthetriae* phenology, combining field observations and laboratory experiments to investigate its ability to overwinter in spongy moth mature eggs or inside the wasp’s cocoons. However, no evidence was found to support this overwintering ability. The findings underscore the reliance of *G. porthetriae* on suitable overwintering hosts, emphasizing the ecological dependency on alternative host species during autumn and winter.

## 1. Introduction

The spongy moth, *Lymantria dispar* (Lepidoptera: Erebidae), is a significant forest pest across many regions worldwide. Its cyclic population outbreaks often lead to extensive defoliation over large forest areas. While *L. dispar* is highly polyphagous, there are considerable regional differences in host plant preferences. Historically, outbreak hotspots in its native range were primarily located in southeastern European oak forests [1]. However, climate change is expected to shift the optimal distribution range of this thermophilous species further north [2]. Spongy moth populations are frequently managed through chemical control measures [1,3], with unintended adverse effects on non-target organisms, raising concerns about their ecological impact [4].

The spongy moth is characterized by a highly diverse complex of natural enemies, which play a pivotal role in regulating its population dynamics [5]. Among these, larval parasitoids are particularly significant, as they possess the potential to delay, suppress, or terminate population outbreaks [6,7]. Despite their ecological importance, many aspects of parasitoid biology remain poorly understood, including critical life cycle processes such as overwintering in temperate climates [8]. Enhancing our understanding of these processes could facilitate the integration of parasitoids into sustainable spongy moth management strategies.

One such parasitoid is the strictly solitary koinobiont endoparasitic wasp *Glyptapanteles porthetriae* (Hymenoptera: Braconidae) [9], native to extensive regions of the Palearctic [10,11]. In spring, females parasitize early instars of *L. dispar* [12,13]. Parasitized host larvae typically succumb during the second to fourth instar, often in the instar following the one attacked. Parasitization significantly prolongs the duration of the final host instar. After egression from the host, the wasp larva spins a white cocoon, pupates, and emerges as adult wasp approximately one week later [14].

Although *G. porthetriae* is widely distributed across Europe, its abundance is typically sporadic, with parasitization rates of *L. dispar* rarely exceeding 10% [15]. The species is most prevalent during periods of low spongy moth density [5] and demonstrates a rapid response to host population growth [12]. This dynamic response suggests that *G. porthetriae* may play a significant role in regulating or preventing outbreaks of this major forest pest [6]. However, at high spongy moth population levels, parasitization rates by *G. porthetriae* are generally low. This limitation is hypothesized to stem from the wasp’s dependence on alternative host species during late summer through the following spring, when the univoltine spongy moth overwinters in the egg stage [1]. The lack of suitable overwintering hosts is believed to constrain the reproductive potential of *G. porthetriae* in spongy moth outbreak populations [16]. This limitation is also considered a key factor in the failure to establish *G. porthetriae* in North America, despite extensive introduction efforts in the early 20th century [12].

Although *G. porthetriae* has been a subject of scientific interest over decades [10], significant aspects of its life cycle, particularly its overwintering biology, remain poorly understood [9]. Field observations suggest that the first instar larvae of *G. porthetriae* within the haemocoel of host larvae serve as the overwintering stage [15]. Several species from the families Erebidae (*Euproctis chrysorrhoea*, *Phragmatobia fuliginosa*, *Diacrisia virginica*, *Orgyia leucostigma*, *Hyphantria cunea*) and Geometridae (*Alcis repandata*, *Peribatodes rhomboidaria*) have been described as potential overwintering hosts [11,15,17,18]. However, most records are based on isolated observations, and the predominant overwintering hosts under natural field conditions remain unidentified.

In addition to overwintering as endoparasitic first instar larvae, overwintering as cocooned prepupae is the most common strategy among microgastrine braconid wasps [19]. *Cotesia melanoscela* (Hymenoptera: Braconidae), a closely related species, can facultatively employ both overwintering strategies [9], whereas most microgastrine species are restricted to a single pathway [19]. Shaw & Skelton [9] hypothesized that *G. porthetriae* is highly specialized, largely univoltine, and potentially overwinters in the mature diapausing egg larvae of *L. dispar*. This hypothesis is supported by the wasp’s immediate presence in an isolated, introduced *L. dispar* population in the U.K. and its specific abdominal adaptations, which are likely suited for egg parasitism. Similar strategies have been documented in *Cotesia glomerata* (Hymenoptera: Braconidae) [20,21] and the Neotropical relative *Glyptapanteles jimmilleri* (Hymenoptera: Braconidae) [22], although such cases are considered exceptional within the Microgastrinae [19].

The ability of insects to survive low winter temperatures in temperate regions typically necessitates a transition to a dormant state. Dormancy may manifest as an immediate response to adverse environmental conditions (quiescence) or as a more complex, endogenously mediated process (diapause). Both forms of dormancy are typically associated with reduced metabolic activity [23]. The respiration rate, as an indirect measure of metabolic activity, can be used to assess diapause intensity [24,25]. For example, in *L. dispar* eggs, the respiration rate of diapausing eggs is 2-fold to 26-fold lower compared to post-diapausing eggs [26].

Dormancy in *G. porthetriae* has not been explicitly studied. Continuous rearing on *L. dispar* under laboratory conditions rules out obligate diapause [10], underscoring the role of exogenous factors. In temperate insects, photoperiod is generally considered the primary cue for inducing winter diapause; however, in many parasitic hymenopterans, temperature and host interactions also play important roles in diapause regulation [27]. While the effects of photoperiod on *G. porthetriae* have not been examined, a linear decrease in the developmental rate of immature stages with temperature has been reported under long-day conditions, with a requirement of 235 degree-days for endoparasitic development, and a lower developmental threshold (LDT) of 6.9 °C [28].

An unusually high abundance of *G. porthetriae* was recorded during a 2020 investigation of the natural enemy complex associated with a declining *L. dispar* outbreak population in Austria [7]. This finding highlights the potential ecological significance of *G. porthetriae* within the spongy moth’s natural enemy complex. Utilizing data from this study, we identified notable variations in the bioecology of *G. porthetriae* under specific weather conditions, emphasizing the critical role of phenological synchronization of parasitoid and host. To further elucidate the species’ ecological adaptations, we conducted a series of laboratory experiments to assess the effects of photoperiod and temperature as potential dormancy-inducing factors on the immature development of *G. porthetriae*. Furthermore, we investigated the parasitoid’s capacity to overwinter independently of alternative host species, either as cocooned prepupae or within *L. dispar* egg larvae.

## 2. Materials and Methods

### 2.1. Study Site

Field investigations were conducted in an isolated mixed oak forest of approximately 100 hectares in Eggenburg, Lower Austria (48°38′20″ N, 15°49′40″ E). The forest is located in the warm-arid Pannonian sub-continental climate zone, with an average annual temperature of approximately 10.0 °C and annual precipitation of 500 mm. Air temperatures during the study period (April to July 2020) were recorded using Hobo^®^ Pendant (UA-002-64; Onset Computer Corporation, Bourne, MA, USA) temperature/light data loggers. The dominant tree species is *Quercus petraea*, with co-occurring species including *Quercus cerris*, *Pinus sylvestris*, *Acer platanoides*, *Robinia pseudoacacia*, *Quercus robur*, and *Prunus avium*.

#### 2.1.1. Evaluation of Host Population Density

A spongy moth outbreak in 2018 caused complete crown defoliation in the forest during 2018 and 2019. Population density estimates for early spring 2020 and 2021 were conducted using the Turček method [29]. Fixed transects were established along cardinal directions radiating from the center of the study plot. Spongy moth egg masses were visually counted on 400 trees (100 trees per transect) up to a height of 4 m, including the trunk, crevices, and branches at the first junction. Each tree was treated as a sampling unit. To ensure accuracy, two independent observers conducted separate counts of fresh, light-brown egg masses (deposited in summer 2019).

#### 2.1.2. Sampling Techniques and Recording of Phenological Data

First to fourth instar *L. dispar* larvae (L1–L4) were collected on five dates between 14 May and 23 June. First and second instars were collected exclusively on 14 May, with second instars also present on two subsequent dates but not sampled further due to sufficient sample size. Early instars were collected from branches using beating nets, third and fourth instars were also collected from tree trunks. Phenological data on larval instars were recorded during each collection.

Larval hatching was monitored from 20 *L. dispar* egg masses collected in early April and stored under semi-field conditions. Data regarding late instars, pupae, adults, and eclosion of *G. porthetriae* wasps were obtained from individuals reared under semi-field conditions. Spongy moth larval instars were identified based on head capsule width [30]. Reference measurements were performed on larvae from egg masses collected at the study site. Approximately 15–20 larvae were reared individually from hatching, and head capsules were collected and measured after each molt using a calibrated stereo microscope (accuracy: 0.01 mm). Each instar exhibited a distinct, non-overlapping range of head capsule widths, which increased progressively with each molt.

#### 2.1.3. Parasitization of *L. dispar* by *G. porthetriae*

Collected larvae were transferred to 250 mL plastic containers, fed oak leaves from the collection site three times per week, and reared under semi-field conditions, protected from rain, until death or adult moth eclosion. Due to the natural aggregation of the young larvae [31], first and second instars were reared in groups of 10 individuals per container. From the third instar, all larvae were reared individually.

Parasitization was assessed by monitoring parasitoid emergence from any host stage. Emergence rates were calculated as the proportion of individuals with parasitoid emergence relative to the total number of collected individuals at each stage. Parasitoid species were identified based on cocoon morphology and verified using adult specimens whenever possible [32]. Cocoons were stored in 1.5 mL Eppendorf tubes with air exchange holes and monitored for wasp eclosion for 60 days. Non-eclosed cocoons were dissected, revealing dead pupae or prepupae. Sex determination was conducted for all adult wasps based on the presence or absence of the ovipositor.

#### 2.1.4. Hyper-parasitization of *G. porthetriae*

To assess hyperparasitism, *G. porthetriae* cocoons were collected from leaves and tree trunks at the study site. A total of 37 cocoons were collected on 28 May and eight cocoons on 2 June. Cocoons from each collection date were stored separately in 250 mL plastic containers. Emerging wasps were counted and identified to the family or subfamily level following the classification guidelines of Broad [33].

### 2.2. Laboratory Experiments on the Overwintering Biology of G. porthetriae

The ability of *G. porthetriae* to develop as an egg–larval parasitoid or enter dormancy as a cocooned prepupa, and the influence of photoperiod and temperature as dormancy-inducing factors, were investigated through laboratory experiments and a semi-field trial. For Experiments 2 and 3, *L. dispar* larvae were obtained from egg masses provided by the USDA/APHIS Otis Method Development Center (Cape Cod, MA, USA), and *G. porthetriae* wasps were sourced from our laboratory colony established from individuals collected in eastern Austria.

#### 2.2.1. Experiment 1—Overwintering of *G. porthetriae* in Field-Collected *L. dispar* Eggs

To determine the presence of *G. porthetriae* in field-collected *L. dispar* eggs, five egg masses were collected from a mixed oak forest (*Quercus cerris*, *Quercus petraea*) in Klingenbach, Burgenland (47°45′43″ N, 16°33′14″ E) in April 2024. This forest has a history of spongy moth outbreaks, with high *G. porthetriae* abundances reported previously [16]. Since 2005, the *L. dispar* population density has remained below 0.1 egg masses per tree.

After hatching, 100 larvae were sampled from each egg mass (500 larvae in total), covering the full range of hatching times (early to late). Larvae were reared in groups of 25 in 250 mL plastic containers at 20 °C under long-day conditions (16L:8D), fed an artificial diet [34], and monitored for parasitoid emergence. Larvae were reared until the fifth instar, as *G. porthetriae* larvae are unable to emerge from fifth or later instars of *L. dispar* [14].

#### 2.2.2. Experiment 2—Overwintering of *G. porthetriae* in Laboratory-Exposed *L. dispar* Eggs

To evaluate the suitability of embryonic *L. dispar* larvae as overwintering hosts for *G. porthetriae*, *L. dispar* eggs were offered to naïve wasp females at three developmental stages: (i) 14 days post-oviposition, (ii) after visually confirmed completed embryonic development (indicated by discoloration from light to dark), and (iii) 14 days post-embryonic development. Egg batches (40 ± 10 eggs per developmental stage) were covered with abdominal hair from *L. dispar* female moths, according to their natural appearance, and placed in detached lids of 2 mL Eppendorf tubes. Each batch was exposed for 72 h to a single female wasp housed in a 5 × 5 cm cage at 20 °C under long-day photoperiod (16L:8D). The experiment was replicated with 10 *G. porthetriae* females, involving 1164 *L. dispar* eggs.

After exposure, eggs were cleaned of abdominal hair and stored at 20 °C (16L:8D) for 30 days, followed by 120 days at 5 °C (8L:16D), and then returned to 20 °C (16L:8D). Hatched larvae were reared to the fifth instar in groups in 250 mL plastic containers and monitored for parasitoid emergence.

#### 2.2.3. Experiment 3—Dormancy Induction in Endoparasitic Larvae or Cocooned Prepupae

To investigate the single effect of photoperiod on dormancy induction in immature wasps, 50 *L. dispar* larvae (L2d3) were offered to *G. porthetriae* females. Larvae were reared individually in glass Petri dishes, fed an artificial diet [34], and divided into two groups maintained at 20 °C under either long-day (16L:8D) or short-day (8L:16D) conditions. Parasitized larvae were monitored for moulting, mortality, and parasitoid emergence.

Respiratory activity, as an indicator of metabolic activity, was measured for eight larvae per group at 0, 7, and 14 days post-parasitization using a volumetric microrespirometer (custom-built, BOKU University, Vienna, Austria) [35]. Additionally, the respiratory activity of *G. porthetriae* pupae within cocoons (*n* = 24) was measured four days post egression from the host.

#### 2.2.4. Experiment 4—Impact of Autumn Conditions on *G. porthetriae* Development in *L. dispar* Hosts Under Semi-Field Conditions

To evaluate the combined effects of short-day photoperiod and low temperatures on *G. porthetriae* development in *L. dispar* hosts, 60 parasitized larvae were exposed to outdoor autumn conditions. Larvae were parasitized three days post moult to the second instar, reared individually in glass Petri dishes, and fed an artificial diet [34].

The larvae were exposed to natural temperature and photoperiod conditions in Vienna, Austria (48°12′36″ N, 16°21′48″ E), protected from rain and direct sunlight. Parasitization and exposure were conducted in three batches: (i) late August (28 August 2023), (ii) mid-September (18 September 2023), and (iii) late September (25 September 2023), with 20 parasitized larvae per batch. Temperature and photoperiod were recorded using a Hobo^®^ Pendant (UA-002-64) temperature/light data logger. Larvae were monitored regularly for parasitoid emergence. Cocoons were left in Petri dishes until wasp eclosion. Non-eclosed cocoons were dissected after 30 days.

### 2.3. Statistical Analysis

All statistical analyses were performed using R (Version 4.4.2), with a significance level of α = 0.05. Categorical variables (e.g., parasitoid emergence rates, premature host mortality, successful host pupation, wasp eclosion rates, and sex ratios) were compared between groups using Fisher’s exact test. For multiple comparisons, *p*-values were adjusted using the Bonferroni correction. Continuous variables (e.g., developmental duration, respiration rates, and temperature data) were analyzed using two-sided *t*-tests or one-way ANOVA. Normality and homogeneity of variances were assessed using the Shapiro–Wilk and Levene’s tests, respectively. Developmental rates as a function of temperature were evaluated using linear regression.

## 3. Results

### 3.1. Field Study—Host Population Density and Phenology of L. dispar

**Figure 1:**
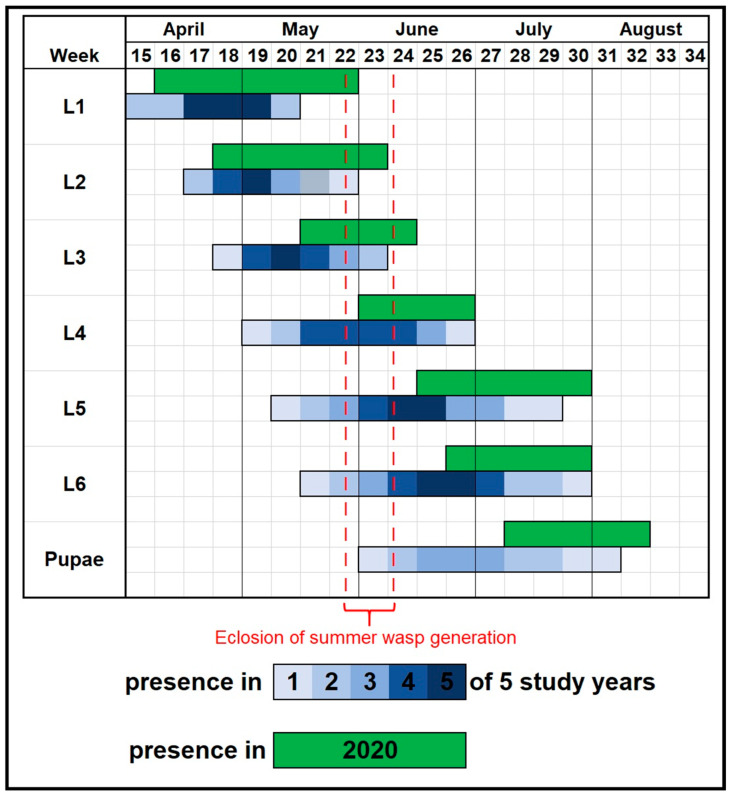
Phenology of *L. dispar* in Eggenburg in 2020 (green) compared with observations from Eastern Austria in 1993–1995 [6,36] and 2003–2004 [37] (blue). Blue bars are shown as a heatmap: stages recorded in only one of the five reference years appear in the lightest blue, while those recorded in all five years appear in the darkest blue. The red segment (calendar weeks 22–24) indicates adult eclosion of the summer generation of *G. porthetriae* in 2020.

The population density of *L. dispar* in Eggenburg was estimated as 13.2 ± 12.6 egg masses per tree in early spring 2020 and collapsed to <0.1 egg masses per tree in spring 2021. The warm spring weather in 2020 resulted in early egg hatching of *L. dispar*. However, a cold spell in May significantly delayed the development of young spongy moth larvae. First instars were observed in the field over a period of seven weeks, second instars for six weeks. By June, temperatures increased significantly, accelerating the development of *L. dispar*. The third and fourth instars progressed rapidly from mid-May to the end of June, with pupation beginning on 6 July. Adult moths emerged between 10 July and the end of August (Figure 1).

#### 3.1.1. Field Study—Emergence Rates of *G. porthetriae* from *L. dispar* Larvae

Stage-specific parasitoid emergence rates of *L. dispar* larvae (L1–L4) in Eggenburg ranged from 15% in first instars to 52% in third instars [7]. *Glyptapanteles porthetriae* was the dominant parasitoid across all instars. In first instars, emergence rates of *G. porthetriae* were significantly lower (10%) compared to the two following instars. The highest emergence rate was observed in second instars (36%), while no significant difference was found between third instars (30%) and second or fourth instars (23%) (Table 1).

The collection date significantly influenced total and stage-specific emergence rates. The highest rates were recorded on 16 June, with *G. porthetriae* emerging from 82% of third instars and 40% of fourth instars (Table 1). On this date, emergence rates for both third (*p* = 0.024) and fourth instars (*p* = 0.018) were significantly higher than for individuals collected on 2 June. Additionally, host instar significantly affected parasitization. Third instars had significantly higher emergence rates than fourth instars on both 2 June (*p* = 0.005) and 16 June (*p* = 0.035).

#### 3.1.2. Field Study—Phenology and Eclosion of *G. porthetriae*

**Figure 2:**
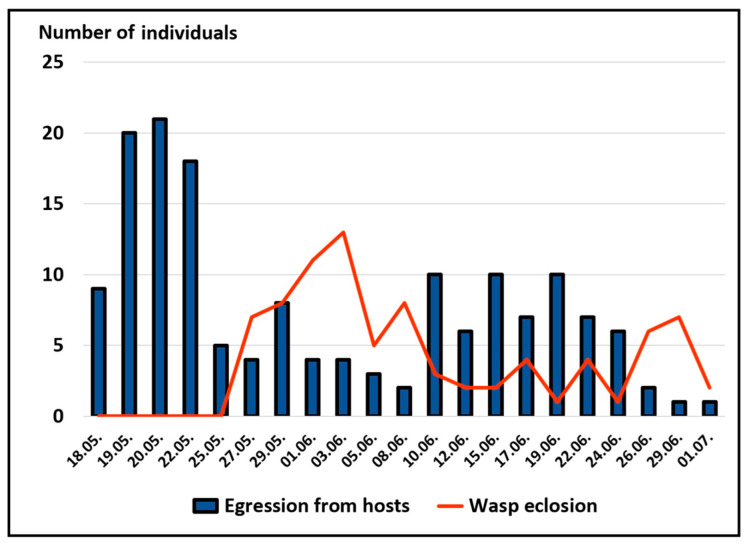
Phenology of *G. porthetriae* in 2020. Blue bars indicate the emergence of *G. porthetriae* larvae from host larvae, and the red line indicates adult eclosion from cocoons.

Mature wasp larvae egressed from second to fourth instar host larvae over a six-week period, from 18 May to 1 July. The timing of egression exhibited a bimodal distribution, with peaks in mid-May and mid-June. Most host larvae (76%) died within the instar in which they were collected, with an average survival time of 9.4 ± 6.5 days post collection. In first instars, survival time (18.3 ± 7.4) was significantly extended (*p* < 0.001) compared to all later instars (6 to 10 days). Adult wasps eclosed 11.1 ± 3.6 days after emerging from the host larvae (27 May to 1 July). A pronounced peak was observed in early June, followed by a smaller peak at the end of June (Figure 2).

Eclosion occurred in a protandric pattern, with the first peak of male emergence on 27 May. The peak in female and overall eclosion was recorded on 3 June, approximately two weeks before the highest emergence rates of *G. porthetriae* were observed in the field (16 June) (Table 1). While early (L1 + L2) and mid (L3 + L4) host instars showed similar emergence rates, significant differences were found in the eclosion from cocoons and the sex ratio (Table 2). Similarly, eclosion rates were significantly higher for *G. porthetriae* individuals emerging from host larvae collected in May compared to those collected in June (61% vs. 38%, *p* = 0.008). However, eclosion rates did not differ between larvae collected in a given instar on different dates. The overall sex ratio of eclosed wasps was 42:58 (F:M) but varied significantly depending on the host instar at collection. Wasps emerging from younger larvae exhibited a balanced sex ratio, whereas the sex ratio of wasps emerging from older larvae was male-biased (Table 2).

Five true hyperparasitoids (attacking the endoparasitic larvae inside the host) emerged from five laboratory-reared *G. porthetriae* cocoons originating from field-collected *L. dispar* larvae. Four of these hyperparasitoids were identified as members of the ichneumon wasp subfamily Mesochorinae, while one individual belonged to the subfamily Cryptinae (Hymenoptera: Ichneumonidae).

Pseudohyperparasitoids, which attack the braconid pupae, caused significantly higher mortality than true hyperparasitoids. The overall eclosion rate of field-collected *G. porthetriae* cocoons was comparable to that of cocoons obtained from field-collected hosts. However, 77% of all eclosed wasps were hyperparasitoids, which emerged significantly later (*p* < 0.001) than *G. porthetriae* (Table 3).

The 20 pseudohyperparasitoid individuals were identified as belonging to at least five species from three hymenopteran families [20]: the chalcidid families Pteromalidae and Eurytomidae, and the ichneumonid subfamily Cryptinae.

### 3.2. Development of G. porthetriae as Egg–Larval Parasitoid

Not a single individual of *G. porthetriae* emerged from *L. dispar* larvae that were exposed to *G. porthetriae* females in the egg stage under field or laboratory conditions. This comprises the 500 *L. dispar* larvae obtained from egg masses collected in Klingenbach (Experiment 1), more than 1000 larvae derived from eggs exposed to *G. porthetriae* in the laboratory (Experiment 2), and the larvae used as head capsule width references originating from eggs collected in Eggenburg. The eggs exposed to *G. porthetriae* in the laboratory exhibited a hatching rate >99%.

### 3.3. Dormancy Induction in Endoparasitic Larvae or Cocooned Prepupae

Parasitization of second instar *L. dispar* larvae under laboratory conditions (Experiment 3) resulted in 64% successful wasp emergence, with no significant difference in response to photoperiod. While 4% of the host larvae died within ten days of parasitization, 32% developed into *L. dispar* pupae without parasitoid emergence. The photoperiod had no significant effect on the duration of endoparasitic or pupal development of *G. porthetriae*, or the respiratory activity of parasitized *L. dispar* larvae and *G. porthetriae* cocoons (Table 4).

**Figure 3:**
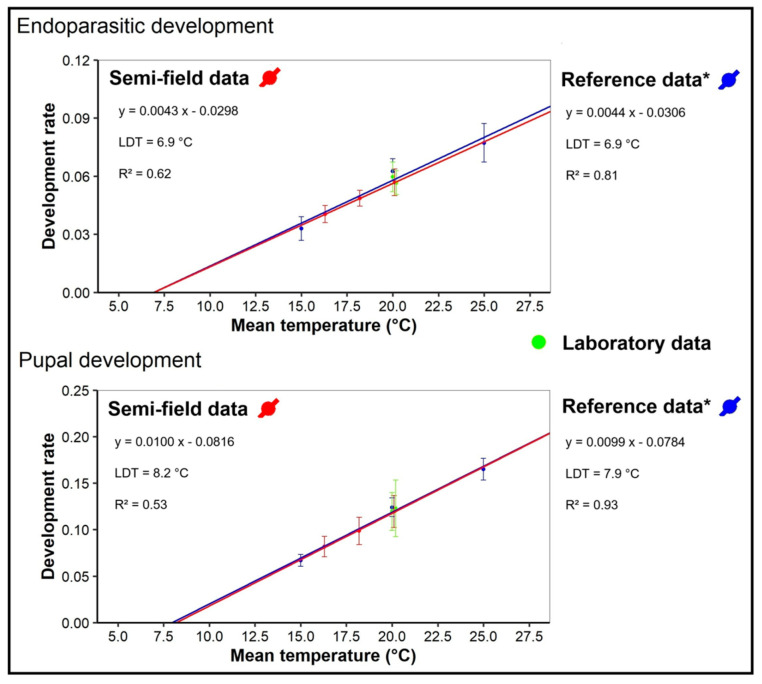
Developmental rate (1/duration) of endoparasitic (top) and pupal (bottom) development of *G. porthetriae* under autumn semi-field conditions (red) (Experiment 4) and under short-day or long-day laboratory conditions (green) (Experiment 3). Reference data* from long-day laboratory experiments at 15 °C, 20 °C, and 25 °C are indicated in blue [28]. Dots show mean values with standard deviation (SD) error bars. Linear regressions and lower developmental thresholds (LDTs) were estimated for the semi-field (red) and reference (blue) datasets and largely overlap. The LDT is the temperature at which the predicted developmental rate equals 0.

In the semi-field trial (Experiment 4), mean temperatures ranged from 16 to 20 °C, while photophases decreased from 13.5 to 10.5 h in the course of the experiment. No significant differences were found in parameters related to parasitization success or the eclosion rate from wasp cocoons (Table 4). The average duration of endoparasitic development and pupal development increased linearly with decreasing temperatures (Figure 3).

## 4. Discussion

Our field study provides new insights into the phenology of *G. porthetriae* and its potential as a key natural enemy of the spongy moth in Central Europe. Notable findings include its dominance among parasitoids of *L. dispar*, high emergence rates from mid-instar hosts, and a prolonged period of larval egression and adult eclosion. The elevated abundance of *G. porthetriae* in the third spongy moth outbreak year led us to test whether it can develop predominantly as a monophagous parasitoid, rather than relying obligatorily on alternative hosts as commonly assumed in the literature.

Earlier field studies reported the dominance of other braconids, such as *Glyptapanteles liparidis* (Hymenoptera: Braconidae) [16,38] and *Cotesia melanoscela* (Hymenoptera: Braconidae) [39,40], or generally low braconid parasitism [37,41,42,43]. In recent decades, *G. porthetriae* has played a negligible role in the decline of European spongy moth populations or was entirely absent [16,37,38,40,42,43,44], with high abundances reported only in southeastern Europe [45,46].

In our study, high *G. porthetriae* abundance appears to have been facilitated by a previously unreported pattern: parasitism of *L. dispar* by two *G. porthetriae* generations. Wasp larvae egressed from hosts over six weeks in a bimodal pattern; the second peak occurred about two weeks after a pronounced peak of adult eclosion, matching the endoparasitic development time of *G. porthetriae* [14]. This divergence from earlier findings likely reflects the specific weather conditions. Early larval hatching was followed by a marked developmental delay and prolonged presence of first- and second-instar spongy moths. Nevertheless, the timing of wasp larval egression in mid-May matched previous reports (±7 days), resulting in the presence of mainly second- and third-instar hosts at the time of wasp eclosion in 2020, compared to fourth or later instars in earlier studies [36,37]. This implies a stronger delay in host development driven by differing thermal requirements of parasitoid and host. Based on lower developmental thresholds (LDTs) for endoparasitic development of *G. porthetriae* (6.9 °C) [28] and larval development of the studied *L. dispar* population (10.1 °C) [47], *G. porthetriae* accumulated a 76% higher effective temperature sum in May 2020. Consequently, early instars remained available as hosts during the adult eclosion of the first *G. porthetriae* generation in early June, whereas previous studies typically reported fourth to sixth instars at that time [6,37].

The preference of *G. porthetriae* for younger *L. dispar* larvae, particularly first and second instars, is well documented. For example, Nussbaumer & Schopf [14] reported parasitization success of 68% in first instars and only 17% in third instars. By contrast, we observed higher emergence rates from third (81%) and fourth instars (40%) in mid-June. This may reflect inverse population dynamics, with declining spongy moth density and rising natural enemy abundance during the second *G. porthetriae* generation. Such imbalance likely increased superparasitism and multi-parasitism [7], both known to enhance parasitization success in less suitable hosts [48,49,50,51]. Nonetheless, reduced adult eclosion and a strongly male-biased sex ratio in individuals emerging from mid instar hosts indicate suboptimal host suitability.

Field data on hyperparasitism of *G. porthetriae* are scarce. Rates of 7% were reported from Serbia in the 1980s during periods of low spongy moth density [45]. Although our sample size precludes precise estimates, our findings suggest substantially higher rates and a density dependent response to increased primary parasitoid abundance. Similarly high hyperparasitism was recorded at the same site in 2019 for *G. liparidis* [52] and may have contributed to the near absence of the typically dominant *G. liparidis* and *C. melanoscela* in 2020. *Cotesia melanoscela* is particularly vulnerable to pseudohyperparasitoids due to the prolonged exposure of its overwintering cocoons [53]. Clusters of gregarious *G. liparidis* cocoons may be easier to locate [54], although survival of some individuals within gregarious broods reduces the per capita risk [55].

No wasp larvae emerged from hosts originating from eggs exposed to *G. porthetriae* in the field or laboratory. Despite the relatively small sample of five field exposed egg masses, we are confident that substantial use of *L. dispar* egg larvae as overwintering hosts would have been detected. *Glyptapanteles porthetriae* has been consistently present at the site, and empty cocoons were regularly observed on tree trunks during egg sampling. Ruberson & Whitfield [56] reported that 25% of *Spodoptera exigua* egg masses and 30–40% of emerging larvae yielded parasitoids for the facultative egg–larval parasitoid *Cotesia marginiventris* (Hymenoptera: Braconidae). Given the much larger *L. dispar* egg masses, an accumulation effect [57] would suggest higher proportions of parasitized egg masses. Egg–larval parasitism is also inconsistent with the higher emergence rates from hosts collected as second instars versus first instars, especially because *G. porthetriae* parasitism delays host development [14]. Second instars had longer larval exposure to parasitism, whereas the prolonged survival of first instars suggests recent parasitization in that group.

Our laboratory results likewise did not support egg–larval development. In Microgastrinae with facultative egg–larval parasitism, females readily show strong interest in host eggs [20,21,56], which we never observed in *G. porthetriae*. Furthermore, females emerging from *L. dispar* in spring would be unlikely to survive the extended interval required for host eclosion, oviposition, and completion of embryogenesis in *L. dispar*. Although this gap can be bridged with noctuid intermediate hosts [58], in turn this weakens the hypothesis of strong specialization on the spongy moth.

Overwintering as an endoparasitic larva or cocooned prepupa requires dormancy induction in immature stages or through maternal mediation [59]. In temperate insects, photoperiod is considered as key cue for winter diapause; in many parasitic Hymenoptera, temperature and host interactions also play important roles [27,60]. Critical photoperiod and temperature thresholds often interact and vary regionally. For European *C. melanoscela*, the photoperiod inducing 50% diapause is ~12.5 h at both 24 °C and 30–35 °C, versus 17 h and 14.5 h in a Connecticut population [61]. In *Cotesia plutellae* (Hymenoptera: Braconidae), the same photophase elicits ~100%, 40%, or 0% diapause at 17 °C, 20 °C, or 25 °C, respectively [62].

Parasitoid dormancy is often regulated by the host’s physiological and endocrine state [63], although interactions are variable and reciprocal. In *Aporia crataegi* (Lepidoptera: Pieridae) (obligate larval diapause), *Cotesia glomerata* and *Cotesia pieridis* (Hymentopera: Braconidae) are forced into obligatory diapause as first instar endoparasites [21,64,65]. In contrast, both species exhibit facultative prepupal diapause after development in *Pieris brassicae*, induced by photoperiod. In *C. glomerata*, induction is host independent and can be triggered even in post diapause *A. crataegi* hosts [65].

When developing in *L. dispar*, the life cycles of the two closest ecological relatives of *G. porthetriae* differ. Both *G. liparidis* and *C. melanoscela* have two generations in *L. dispar*, but the plurivoltine *G. liparidis* releases adult wasps in summer [6], whereas overwintering as a cocooned prepupa predominates in the bivoltine *C. melanoscela* [12,61,66]. However, both species show similar patterns in *E. chrysorrhoea* and *Leucoma salicis* (Lepidoptera: Erebidae), hosts with effectively obligate larval diapause in nature. Parasitism of early prediapause instars in autumn leads to parasitoid larval dormancy; parasitism of post diapause hosts in spring results in direct development [67,68,69]. In prediapause hosts, dormancy induction in *G. liparidis* is obligate and environment-independent [69], whereas in *Dendrolimus pini* (Lepidoptera: Lasiocampidae) (facultative larval diapause), *G. liparidis* dormancy incidence is photoperiod-dependent [30,70]. Comparable host switching life cycles and dormancy types occur in other oligophagous braconids, e.g., *Meteorus versicolor* with *E. chrysorrhoea*/*L. dispar* & *Thaumetopoea processionea* or *Diprion pini*/*Panolis flammea* as overwintering/summer hosts [71], and *Apanteles lateicolor* using *E. chrysorrhoea*/*L. dispar* [72].

In overwintering hosts, direct developmental suppression tied to host diapause appears to be the main driver of parasitoid larval dormancy; in summer hosts, regulation is more complex. For *P. brassicae*/*C. glomerata*, parasitoid diapause induction can be host independent. Other summer hosts mentioned, including *L. dispar*, are univoltine with obligate diapause in eggs or pupae [30,65]. Assuming such hosts are insensitive to diapause-inducing cues, dormancy likely depends on autonomous parasitoid sensitivity.

In our experiments, multiple developmental stages of *G. porthetriae* and *L. dispar* experienced a wide range of temperatures and photoperiods, with no evidence of dormancy induction under any condition. We therefore exclude an independent dormancy induction mechanism as in *C. glomerata*. However, preliminary work with potential overwintering hosts showed a strong photoperiodic response in endoparasitic development when *G. porthetriae* develops in *Lasiocampa quercus sicula* (Lepidoptera: Lasiocampidae) [73]. Although diapause in this host is regularly induced in larvae but initiated only after pupation [65], parasitized hosts showed pronounced suppression of larval development in response to photoperiod. This indicates a mutual host–parasitoid interaction not reflected in the examples above. We conclude that dormancy induction in *G. porthetriae* is not solely host driven but requires sensitivity to induction cues present in its host. Because *L. dispar* does not provide such cues [65], we propose an obligate dependence on alternative hosts.

## 5. Conclusions

Our experiments provide valuable insights into the life cycle of the spongy moth natural enemy *G. porthetriae*. The field study demonstrated how specific weather conditions facilitated the occurrence of a second summer generation in *L. dispar* hosts, a phenomenon not previously observed. However, due to the low suitability of mid-instar *L. dispar* larvae as hosts, this strategy does not appear advantageous at the population level, as it resulted in a high proportion of male wasps in summer. Despite this, unexpectedly high emergence rates were achieved in third and fourth spongy moth instars. This is likely due to the significant, yet often underestimated, effects of superparasitism and multi-parasitism in *L. dispar* field populations. The observed decline in eclosion rates of *G. porthetriae* cocoons in June was most likely a consequence of the reduced quality of older host larvae rather than from environmental factors that induced dormancy.

Our laboratory and semi-field experiments provided no evidence that *G. porthetriae* is able to overwinter in *L. dispar* egg larvae or as cocooned prepupa. Although certain potentially influential factors, such as maternal effects—documented in *Cotesia vestalis* (Hymenoptera: Braconidae) [59]—or extreme temperatures, have not been explicitly examined, our field investigations partially addressed these variables and showed no significant effects. Consequently, our results support the prevailing hypothesis that *G. porthetriae* relies on alternative host species to enable overwintering of its endoparasitic larvae. However, a high degree of plasticity in dormancy mechanisms of a species is possible in response to environmental conditions [74] or the availability of hosts [21,64,65] and bioecology may vary regionally.

Since environmental factors, particularly photoperiod, had only a minimal influence on the development of *G. porthetriae* in *L. dispar*, we propose that host–parasitoid interactions play a crucial role in inducing *G. porthetriae* dormancy. Future research will therefore focus on identifying potential overwintering hosts with different dormancy strategies to improve our understanding of the host–parasitoid interactions that govern the life cycle and overwintering mechanisms of *G. porthetriae*.

## Figures and Tables

**Table 1:** Emergence rates (%) of *G. porthetriae* larvae from different instars of *L. dispar* collected in Eggenburg during May–June 2020. Values in parentheses are the absolute numbers of emerged wasp larvae by collection date and host instar. Uppercase letters compare the same instar across collection dates; different letters indicate significant temporal differences in emergence rates. Lowercase letters compare instars within a given collection date; different letters indicate significant differences among instars at that time point. Frequencies were compared using Fisher’s exact test, with Bonferroni adjustment for multiple comparisons.

Instar	14 May	26 May	2 June	16 June	23 June	Total
**L1**	10.1 (10) ^a^	-	-	-	-	10.1 (10) ^a^
**L2**	35.7 (85) ^b^	-	-	-	-	35.7 (85) ^b^
**L3**	-	^A^ 18.0 (9)	^A^ 30.9 (27) ^a^	^B^ 81.8 (9) ^a^	-	30.2 (45) ^b^
**L4**	-	-	^A^ 8.9 (4) ^b^	^B^ 40.0 (14) ^b^	^AB^ 25.0 (1)	22.6 (19) ^ab^
**L1–L4**	^A^ 28.2 (95)	^A^ 18.0 (9)	^A^ 23.3 (31)	^B^ 50.0 (23)	^AB^ 25.0 (1)	28.1 (160)

**Table 2:** Emergence from host larvae, eclosion from cocoons, and sex ratio of *G. porthetriae* obtained from *L. dispar* larvae collected at early or mid instars. Frequencies were compared using Fisher’s exact test.

	Early Instars (L1 + L2)	Mid Instars (L3 + L4)	*p*-Value
Parasitoid emergence rate (%)	28	27	0.924
Wasp eclosion rate (%)	63	39	0.004
Sex ratio (F:M)	50:50	23:77	0.042

**Table 3:** Eclosion rates and time from collection to eclosion of field-collected *G. porthetriae* cocoons.

	*G. porthetriae*	Hyperparasitoids	Total
Wasp eclosion rate (%)	13	44	58
Days from collection to eclosion	6.3 ± 6.0	28.5 ± 5.1	

**Table 4:** Parasitization success and developmental parameters of *G. porthetriae* developing in *L. dispar* larvae under constant 20 °C with long- or short-day photoperiods (Experiment 3) (top), or natural temperature and photoperiod conditions in autumn 2023 (Experiment 4) (bottom). Categorical variables were compared using Fisher’s exact test, continuous variables were compared with one-way ANOVA/Tukey test. Different lowercase letters within a row denote significant differences in post hoc comparisons at *p* < 0.05.

**Laboratory Trial**	**16L:8D**	**12L:12D**		***p*** **-Value**
Emergence rate (%)	72	56		0.377
Eclosion rate (%)	100	100		1.000
Premature host death (%)	0	8		0.490
Host pupation (%)	28	36		0.762
Endoparasitic dev. (days)	17.1 ± 2.3	17.6 ± 1.9		0.296
Pupal development (days)	8.5 ± 1.8	8.3 ± 2.1		0.772
Respiration (µL O_2_ mg^−1^ h^−1^)	0.81 ± 0.42	0.83 ± 0.35		0.881
**Semi-Field Trial**	**Early Sep**	**Mid Sep**	**Late Sep**	***p*** **-Value**
Emergence rate (%)	65	55	70	0.709
Eclosion rate (%)	92	100	91	0.522
Premature host death (%)	20	15	10	0.900
Host pupation (%)	15	30	20	0.630
Endoparasitic dev. (days)	17.8 ± 2.2 ^a^	20.7 ± 1.8 ^b^	25.0 ± 2.9 ^c^	< 0.001
Pupal development (days)	8.5 ± 1.3 ^a^	10.4 ± 1.6 ^b^	12.5 ± 1.8 ^c^	< 0.001
Mean temperature (°C)	20.1 ± 4.2 ^a^	18.2 ± 3.4 ^b^	16.3 ± 3.7 ^c^	< 0.001
Photoperiod (hours)	13.5–12.5	12.5–11.0	12.0–10.5	

## Data Availability

The original contributions presented in the study are included in the article. Further inquiries can be directed to the corresponding author.

## Abbreviations

The following abbreviations are used in this manuscript:

APHIS	Animal and Plant Health Inspection Service
L2d3	three days post moult to the second instar
LDT	Lower Developmental Threshold
USDA	United States Department of Agriculture
